# Systematic Review of Plasmid AmpC Type Resistances in *Escherichia coli* and *Klebsiella pneumoniae* and Preliminary Proposal of a Simplified Screening Method for *ampC*

**DOI:** 10.3390/microorganisms10030611

**Published:** 2022-03-14

**Authors:** Enrique Rodríguez-Guerrero, Juan Carlos Callejas-Rodelas, José María Navarro-Marí, José Gutiérrez-Fernández

**Affiliations:** 1 Laboratory of Microbiology, Virgen de las Nieves University Hospital & ibs.Granada—Instituto de Investigación Biosanitaria de Granada, Avda. de las Fuerzas Armadas 2, 18014 Granada, Spain; enriquerg83@gmail.com (E.R.-G.); josem.navarro.sspa@juntadeandalucia.es (J.M.N.-M.); 2 Department of Microbiology, School of Medicine, University of Granada & ibs.Granada—Instituto de Investigación Biosanitaria de Granada, Avenida de la Investigación 11, 18016 Granada, Spain; juancacr97@gmail.com

**Keywords:** plasmidic AmpC betalactamase, *Enterobacteriaceae*, multi-resistant bacteria diagnosis, screening

## Abstract

Beta-lactamase (BL) production is a major public health problem. Although not the most frequent AmpC type, AmpC-BL is increasingly isolated, especially plasmid AmpC-BL (pAmpC-BL). The objective of this study was to review information published to date on pAmpC-BL in *Escherichia coli* and *Klebsiella pneumoniae,* and on the epidemiology and detection methods used by clinical microbiology laboratories, by performing a systematic review using the MEDLINE PubMed database. The predictive capacity of a screening method to detect AmpC-BL using disks with cloxacillin (CLX) was also evaluated by studying 102 *Enterobacteriaceae* clinical isolates grown in CHROMID ESBL medium with the addition of cefepime (FEP), cefoxitin (FOX), ertapenem (ETP), CLX, and oxacillin with CLX. The review, which included 149 publications, suggests that certain risk factors (prolonged hospitalization and previous use of cephalosporins) are associated with infections by pAmpC-BL-producing microorganisms. The worldwide prevalence has increased over the past 10 years, with a positivity rate ranging between 0.1 and 40%, although AmpC was only detected when sought in a targeted manner. CMY-2 type has been the most prevalent pAmpC-BL-producing microorganism. The most frequently used phenotypic method has been the double-disk synergy test (using CLX disks or phenyl-boronic acid and cefotaxime [CTX] and ceftazidime) and the disk method combined with these inhibitors. In regard to screening methods, a 1-µg oxacillin disk with CLX showed 88.9% sensitivity, 100% specificity, 100% positive predictive value (PPV), 98.9% negative predictive value (NPV), and 98.9% validity index (VI). This predictive capacity is reduced with the addition of extended-spectrum beta-lactamases, showing 62.5% sensitivity, 100% specificity, 100% PPV, 93.5% NPV, and 94.1% VI. In conclusion, there has been a worldwide increase in the number of isolates with pAmpC-BL, especially in Asia, with CMY-2 being the most frequently detected pAmpC-BL-producing type of microorganism. Reduction in its spread requires routine screening with a combination of phenotypic methods (with AmpC inhibitors) and genotypic methods (multiplex PCR). In conclusion, the proposed screening technique is an easy-to-apply and inexpensive test for the detection of AmpC-producing isolates in the routine screening of multidrug-resistant microorganisms.

## 1. Introduction

The loss of susceptibility to beta-lactam antibiotics in Gram-negative bacteria is an emerging problem worldwide and is mainly attributable to the production of beta-lactamases, especially extended-spectrum (ESBL) and AmpC type (AmpC-BL) beta-lactamases and carbapenemases [1]. The CESPM group (*Citrobacter freundii*, *Klebsiella aerogenes*, *Enterobacter cloacae*, *Serratia marcescens*, *Providencia stuartii,* and *Morganella morganii*) comprises common *Enterobacteriaceae* species responsible for nosocomial and community infections produced by inducible chromosomal AmpC beta-lactamases. However, other more prevalent species in patients, such as *Escherichia coli* and *Klebsiella pneumoniae*, can present these enzymes with plasmid gene encoding (pAmpC-BL). There has been limited research on pAmpC-BL in comparison to the more frequently detected ESBL- and carbapenemase-producing microorganisms [1,2,3]. There has been no estimate of the true worldwide prevalence of pAmpC-BL-producing microorganisms [4], and no consensus on the most effective laboratory technique for their detection [5]. AmpC-BL-producing microorganisms can also be associated with other types of resistance, highlighting the coexistence of AmpC-BL and ESBL [3].

Colonization of the intestine and larynx may serve as an important reservoir for resistance genes [6,7] of the microorganisms that inhabit them, and can be a risk factor for infection. There is a need for simple, effective, easy-to-apply, and inexpensive techniques to screen for these pathogens in the digestive tract of infected or colonized patients. ESBL-producing *Enterobacteriaceae* colonies can be detected by various techniques, including the use of CHROMID ESBL (bioMérieux, France). This transparent medium contains cefpodoxime and other substances that inhibit Gram-positive bacteria growth, and chromogenic substrates that presumptively identify genera and species according to their color (pink/burgundy for *E. coli*; blue/green for *Klebsiella*, *Enterobacter*, *Serratia*, or *Citrobacter*; and light to dark brown for *Proteae*) [8]. The inclusion of cefoxitin (FOX), cefepime (FEP), and ertapenem (ETP) disks on CHROMID ESBL medium has been proposed for the presumptive identification of ESBL- and/or carbapenemase-producing microorganisms through their resistance to these antibiotics, and a halo diameter breakpoint of 16 mm has proven diagnostically useful [9]. However, the diagnostic performance can be further improved by the addition of other antibiotic disks to reveal the possible presence of AmpC-BL. The production of carbapenemase, ESBL, and AmpC-BL is frequently studied in episodes of colonization by multi-resistant Gram-negative bacteria, and the addition of ETP, FOX, FEP, and cloxacillin (CLX) disks to this medium may offer a simple and effective method for this purpose. CLX shows greater activity against AmpC-BL-producing Enterobacterales but lesser activity against carbapenemase- or ESBL-producing Enterobacterales or carbapenemase-producing *Pseudomonas* spp. and *A. baumannii;* hence, CLX disks may be useful to detect the presence of microorganisms with AmpC [10].

Tests using cultures for the detection of colonies of resistant microorganisms offer an advantage over PCR tests because they detect viable microorganisms and facilitate their recovery, avoiding their loss. Unfortunately, no commercial culture tests are available for the simultaneous detection of Gram-negative microorganisms with different mechanisms of resistance to β-lactam antibiotics. The objectives of the present study were: carry out a systematic review of epidemiological information on pAmpC-BL in *K. pneumoniae* and *E. coli* species; and evaluate the behavior of microorganisms with AmpC-BL in the ChromID^®^ ESBL medium by using the disk diffusion test with FOX, FEP, ETP, and CLX disks.

## 2. Material and Methods

### 2.1. Systematic Review

The PubMed^®^ database was searched using the search term “(AmpC [Title/Abstract]) AND (Plasmid [Title/Abstract])”. Review inclusion criteria were: (i) study on the conceptualization of AmpC type resistances; (ii) study on the epidemiology and clinical relevance of these resistances from 2010 onwards; and (iii) study on their detection. Exclusion criteria were: (i) study of isolation of bacterial species other than *K. pneumoniae or E. coli*; (ii) study of their isolation in food production chains, animals, farms, aqueous media, and other environmental settings; (iii) language other than English or Spanish; and iv) inability to access the text. The search yielded 1001 publications published up to 7 January 2021; 395 of these met the eligibility criteria, and 149 were finally included in the review.

### 2.2. Behavior of Enterobacteriaceae with AmpC-BL in ChromID^®^ ESBL Medium, Using the Disk Diffusion Test with CLX Disks

A retrospective study was conducted in the Microbiology Department of our hospital in Granada (Spain), which covers a population of around 440,000 inhabitants. It included all Enterobacterales strains (67 from rectal swabs and 35 from urine cultures) detected during February 2021 with suspicion of colonization or urinary tract infection by ESBL or carbapenemase microorganisms in individuals aged >14 years). Species were grouped according to their resistance mechanisms, as defined by EUCAST 2021 criteria [11], finding: 47 with ESBL (25 *E. coli*, 19 *K. pneumoniae*, 1 *C. freundii*, 1 *Proteus mirabilis*, and 1 *Klebsiella oxytoca*); 39 with the carbapenemases oxacillinase (OXA) (16 *K. pneumoniae*, 11 *E. cloacae*, and 1 *E. coli*), *Klebsiella pneumoniae* carbapenemase (KPC) (*6 K. pneumoniae*), and Verona integron-encoded metallo-beta-lactamase (VIM) (5 *K. pneumoniae*); 9 with AmpC (5 *M. morganii*, 2 *Kluyvera intermedia*, 1 *E. cloacae*, and 1 *K. aerogenes*); and 7 with ESBL and AmpC (*6 E. cloacae* and 1 *K. pneumoniae*).

Isolates were identified using the MicroScan system (Beckman Coulter, Brea, CA, USA) and mass spectrometry (Maldi-Tof^®^, Bruker Daltonik GmbH, Bremen, Germany). Resistances were characterized with the MicroScan microdilution system, followed, when appropriate, by carbapenemase determination using the Rapidec^®^ Carba NP colorimetric test (BioMerieux, Marcy l’Etoile, France) and immunochromatography (NG5-Test Carba, NG Biotech, Guipry, France). The carbapenemase-producing type was confirmed by the Andalusian Molecular Typing Laboratory of the Spanish PIRASOA Program using mass sequencing (Illumina Inc, San Diego, CA, USA), CLC Genomics Workbench v10 software (Qiagen), ResFinder (Lyngby, Denmark) (https://cge.cbs.dtu.dk/services/ResFinder, (accessed on 30 November 2021)), and CARD (Hamilton, ON, Canada) (https://card.mcmaster.ca/, (accessed on 30 November 2021)) databases. ESBL production was defined by resistance to cefotaxime (CTX) and/or ceftazidime (CAZ) and synergy with clavulanic acid (CLAV) and FEP, and by susceptibility to amoxicillin/CLAV, piperacillin/tazobactam, FOX, and carbapenems. AmpC production was defined by synergy with cloxacillin [3] (with gradient test, cefotetan/cefotetan-CLX E-Test (CTT/CXT), Liofilchem^®^—MIC Test Strip Technical Sheet AmpC), resistance to FOX, amoxicillin/clavulanic acid/CLAV, piperacillin/tazobactam, and CTX and/or CAZ, with an increased minimal inhibitory concentration (MIC) in the presence of CLAV and susceptibility to FEP and carbapenems.

A 0.5 McFarland suspension of each isolate was prepared from colonies grown on lamb blood agar (Becton Dickinson, Franklin Lakes, NJ, USA). Next, a sterilized swab was soaked with the homogenized suspension, excess liquid was removed, and it was uniformly seeded on one half of the plate on CHROMID ESBL medium, streaking the bacterial load on the other half with a calibrated inoculation loop. FEP (30 µg, Becton Dickinson), FOX (30 µg, Becton Dickinson), ETP (10 µg, Becton Dickinson), and CLX (in two variants [test 1 and test 2] to detect AmpC) disks were then placed equidistantly on the seeded area for growth/inhibition measurement with a separation of 1.5 cm between each. The medium was then incubated at 37 °C, with readings at 24 h. Test 1 used a sterile paper disk (BBL ^TM^ TAXO ^TM^ Blank Paper Disks, Becton Dickinson) with a diameter of 13 mm located at the center of the plate with the addition of 20 µL CLX (50 mg/mL) (Sigma-Aldrich, Madrid, Spain); and Test 2 used a disk with 1 µg oxacillin (Becton-Dickinson) with addition of 10 µL CLX (50 mg/mL). IBM SPSS Statistics 19 and Microsoft Excel 2019 were used for statistical analyses. Calculations were made of the diagnostic value of the presence of synergy when applying Tests 1 and 2.

## 3. Results

### 3.1. Systematic Review

#### 3.1.1. Worldwide AmpC-BL Epidemiology

Three AmpC-BL categories have been described: chromosomal type AmpC-BL with inducible expression; chromosomal type AmpC-BL with stable derepression and non-inducible expression (enzyme hyperproduction by mutations in AmpC regulating genes); and plasmid type AmpC-BL (encoded by genes in transfer plasmids) (12). The former enzymes are expressed constitutively and at low concentrations in *Citrobacter* spp., *Enterobacter* spp., *Serratia* spp., *Morganella* spp., and *Providencia* spp. (CESPM group), and in *Pseudomonas aeruginosa* [5,12,13]. However, exposure of these bacterial species to certain beta-lactams can lead to hyperexpression of the encoding gene and elevated production of cAmpC-BL, with the expression of AmpC-BL being inducible. This is attributed to mutations that affect the enzyme responsible for regulating the AmpC-BL gene [3]. Its constitutive expression at low concentrations has also been documented in other bacteria, such as *E. coli*, and in *Shigella* spp., but it is not inducible in these cases because the chromosomal genes of the enzyme lack the natural promoter (*ampR*). Nonetheless, Pfeifer et al. (2010) described cases of resistance to cephalosporins in *E. coli* mediated by the inducible expression of cAmpC-BL, caused by mutations that increased expression of the enzyme [14]. The rise in plasmid type AmpC-BL over the past few years has been described as an epidemic by some authors [12]. In 1989, it was discovered that *ampC* genes may be transmittable by plasmids [4,15], after the finding in South Korea of an isolate highly resistant to FOX, designated plasmid CMY-1 [15]. pAmpC-BLs have traditionally been described in *Enterobacteriaceae* and other Gram-negative bacilli [3,5] (Table 1). The various plasmid AmpC families have been grouped as follows: CIT group of *C. freundii* (including LAT- and some CMY-, such as CMY-2 and BIL); EBC group of *Enterobacter* spp. (MIR-1, ACT-1); DHA group of *M. morganii* (DHA-1, DHA-2); ACC group of *H. alvei* (highlighting ACC-1); MOX group of *Aeromonas* spp. (MOX- and the rest of CMY); and FOX group, observing a very close genetic relationship between these plasmids and their chromosomal origins [4,16].

Species that can express pAmpC-BL include *K. pneumoniae*, *Salmonella*, *E. coli*, *P. mirabilis*, and *C. freundii*, in which these genes are constitutively expressed at high concentrations [15]. All pAmpC-BLs are expressed constitutively, except for DHA-1, ACT-1, DHA-2, and CMY-13 enzymes. These have been described as inducible because the plasmids that contain them include not only the encoding *ampC* genes for the enzyme but also ligated *ampR* genes. These genes are transcription factors responsible for decreasing or increasing the expression of inducible *ampC* genes depending on the cofactor that interacts with AmpR [3,5,13,15,17,18]. Since the discovery of this type of plasmid, it has been repeatedly observed that the most frequently recorded *ampC* gene worldwide is CMY-2 [7,14,17,18,19,20], followed by DHA-1 [18]. Enzymes in the CIT group (CMY-*like*) are predominant in *E. coli*, while enzymes of the DHA family predominate in *Klebsiella* spp. [19,20,21,22,23].

As in the case of Gram-negative bacteria families, AmpC-BL-producing microorganisms produce various types of infection, both nosocomial and community-acquired: urinary tract infections (UTIs, *E. coli* being the most frequently isolated pathogen in this type of infection), intra-abdominal infections, pneumonias, and soft tissue infections, among others [23]. The literature describes various risk factors associated with infections by pAmpC-BL-carrier microorganisms, observing that they do not differ from those described for ESBLs [24], associating AmpC-BL acquisition with previous hospitalization even more than ESBL acquisition [25].

Independent risk factors have been reported for infection by pAmpC-BL-producing microorganisms, including the previous receipt of fluoroquinolones [2,26,27] and cephalosporins such as cephamycin and FOX [2,3,26,27,28], demonstrating the possible therapeutic failure of using cephalosporins as empirical treatment (30) even being considered as an independent risk factor [2,25]. In addition, not all AmpC plasmid families are associated with the same mortality rate; thus, according to the report of Pai et al. (2004), infections produced by AmpC-BL DHA-1 have a higher mortality rate than those produced by CMY-2 [29]. Other risk factors are prolonged hospitalization [2,3,4,28,30] or patient institutionalization (in the study by Rodríguez-Baño et al. (2015)) [25], more than 50% of infections were associated with community outbreaks, especially in patients with associated health care), hospitalization in intensive care units (ICUs), and use of central and urinary catheters [3,25,28], mainly in the case of nosocomial infections. Age, presence of diabetes mellitus, hospital admission, institutionalization in care homes, and the use of urinary catheters were associated with community-acquired infections [2].

The worldwide epidemiology of pAmpC-BLs was evaluated by classifying studies according to their target population in the following three groups: hospital (isolates of patients admitted to hospital centers, including ICUs and health institutions), community (isolates from community sampling, studies performed in primary care, infections acquired in the community, and non-hospitalized patients), and hospital and community. The prevalence of AmpC in the reviewed literature was evaluated by considering the total number of isolates in each study. Table 2, Table 3, Table 4 and Table 5 display data were obtained from Europe, America, Africa, and countries in Asia, Oceania, and the Middle East, respectively.

PRESENCE IN EUROPE (Table 2). Major regional differences can be observed in the percentage of pAmpC-BL positivity. It has remained relatively low in Europe over the past 10 years, ranging from 0.06% Denmark (2010) [31] to 2.6% in Holland (2014) [30,32]. However, higher percentages of positivity have occasionally been observed, such as Holland in 2012 with 5% [33], Spain in 2018 with 14.2% [34], and Germany in 2020 with 11.9% positivity. These higher positivity rates may result from differences in screening method or study population (36). For instance, resistance of clinical isolates to carbapenems was used in the 2018 Spanish study [34] while the 2012 study in Holland evaluated isolates with reduced susceptibility to FOX [33]. pAmpC-BL positivity in *E. coli* is generally less frequent in Europe than that observed in other parts of the world, with percentages of 0.06% reported in Denmark (2010) [31], 0.46% in France (2010) [35], 0.73% in Holland and Germany (2017) [36], 1.28% in Portugal (2019) [37], and 2.4% in Holland (2018) [38]. The prevalence was slightly higher (7.55%) in the study by Findlay et al. (2020) in England, because all of the clinical isolates evaluated showed CTX resistance, used as a screening method [39]. An elevated prevalence of pAmpC-BL was also obtained in Ireland (19%) because the isolates had a AmpC phenotype (positivity in the phenotypic detection procedure) [40]. In the same way, the elevated percentage (12.5%) described in Switzerland (2013) [41] was obtained in isolates with resistance to third-generation cephalosporins. Reports on the positivity of *K. pneumoniae* isolates have varied among European countries, with findings of 0.5% in France (2012) [42], 0.47% in Holland (2012) [33], and 1.04% in Portugal (2019) [37].

**Table 2:** Epidemiology of pAmpC-BLs in Europe (2010–2020).

Author (Reference)	Year of Population Study	Year of Publication	Country of Target Population	Population (H/C) ^a^	Specific Conditions	n	AmpC (%) ^b^		Global ^c^	Genetic Identification	Most Frequent AmpC Enzymes
							*E. coli*	*K.pneumoniae*			
Jørgensen et al. [31]	2006	2010	Denmark	H	ECI	74	0.06	-	-	PCR/WGS	CMY-2
Courpon-Claudinon et al. [35]	2005	2010	France	H	3GCR	1051	0.46	-	-	PCR/WGS	CMY-2
Illiaquer et al. [42]	2007–2009	2012	France	H	KPI	1505	-	0.50	-	PCR/WGS	DHA-1
Voets et al. [33]	2009	2012	Holland	C	ESBL	636	3.93	0.47	5.03	PCR/WGS	CMY-2
Miró et al. [43]	2009	2013	Spain	H	EI	100,132	0.69	1.02	0.64	PCR/WGS	CMY-2
Seiffert et al. [41]	2011	2013	Switzerland	H/C	ECI	611	12.50	-	-	PCR/WGS	CMY-2
Gude et al. [44]	2008–2010	2013	Spain	H	EI	-	-	-	0.56	PCR/WGS	CMY-2
Galán-Sánchez et al. [45]	2011–2012	2014	Spain	H/C	ECI	-	0.78	-	-	PCR/WGS	CMY-2
Reuland et al. [30]	2007	2014	Holland	H	3GCR	503	-	-	2.60	PCR	CMY-2
Jones-Dias et al. [46]	2004–2008	2014	Portugal	H	3GCR	124	-	-	0.80	PCR/WGS	CMY-2
Reuland et al. [47]	2011	2015	Holland	C	EI	550	1.30	-	-	PCR	CMY-2
Ibrahimagić et al. [48]	2009–2010	2015	Bosnia and Herzegovina	H/C	ESBL	85	-	-	8.23	PCR	CMY-2
Alonso et al. [49]	2010–2011	2016	Spain	H/C	ECI	21,563	1.10	-	-	PCR/WGS	CMY-2
Li et al. [40]	2011–2012	2015	Ireland	H	3GCR	95	19	-	-	PCR/WGS	CIT group
Pascual et al. [50]	2010–2011	2016	Spain	H/C	3GCR	841	2.02	-	-	PCR/WGS	CMY-2
Zhou et al. [36]	2012–2013	2017	Holland/Germany	H/C	EI	1087	0.73	-	-	PCR/WGS	CMY-2
Gómara et al. [34]	2013–2014	2018	Spain	H	CR	63	-	-	14.2	PCR	CIT group
Den Drijver et al. [38]	2013–2016	2018	Holland	H	EI	2126	2.40	-	-	PCR	CMY-2
Ribeiro et al. [37]	2010–2016	2019	Portugal	H	3GCR	1246	1.28	1.04	2.60	PCR/WGS	DHA-1
Findlay et al. [39]	2017–2018	2020	England	C	3GCR	225	7.55	-	-	PCR/WGS	DHA-1
Rohde et al. [51]	2014–2015	2020	Germany	C	3GCR	828	-	-	11.90	PCR/WGS	CMY-2

^a^ Type of population studied: Hospital (H)/Community (C). ^b^ Percentage positivity for AmpC among all isolates evaluated in the study. ^c^ Percentage global positivity that includes species other than *E. coli* and *K. pneumoniae* and/or does not differentiate between cAmpC-BL and pAmpC-BL. PCR: polymerase chain reaction; WGS: whole genome sequencing; ECI: *E. coli* isolates; 3GCR: third-generation cephalosporin-resistant; EI: *Enterobacteriaceae* isolates; KPI: *K. pneumoniae* isolates; ESBL: extended spectrum beta-lactamase; CR: carbapenemase resistant.

PRESENCE IN AMERICA (Table 3). Our search of the literature retrieved few publications on the prevalence of pAmpC-BL in America. In general, the global prevalence of pAmpC-BL has been relatively low in the USA, ranging between 1.3% in 2016 [32] and 3.42% in 2019 [52]. Reports on the prevalence of pAmpC-BL in *E. coli* have ranged widely between 2.23%, as described by Tamma et al. (2019) [52], and 16.33%, reported by Park et al. (2012), who studied the presence of pAmpC-BL in FOX-resistant isolates [53]. In Mexico, Paniagua-Contreras et al. (2018) found a higher prevalence (23.7%) of AmpC-BL among *E. coli* isolates [54].

**Table 3:** Epidemiology of pAmpC-BLs in America (2010–2020).

Author (Reference)	Year of Population Study	Year of Publication	Country of Target Population	Population (H/C) ^a^	Specific Conditions	n	AmpC (%) ^b^		Global ^c^	Genetic Identification	Most Frequent AmpC Enzymes
							*E. coli*	*K.pneumoniae*			
Park et al. [53]	2008–2012	2012	USA	H	3GCR	300	16.33	-	-	PCR/WGS	CMY-2
Suwantarat et al. [32]	2014–2015	2016	USA	H	EI	854	-	-	1.30	PCR/WGS	CMY-2
Logan et al. [55]	2011–2015	2016	USA	H	MDR	225	14.22	-	-	PCR/WGS	CMY-2
Paniagua-Contreras et al. [54]	*Data not available*	2018	Mexico	C	ECI	194	23.70	-	-	PCR	CIT group
Tamma et al. [52]	2014–2015	2019	USA	H	EI	1,929	2.23	0.88	3.42	PCR	CMY-2

^a^ Type of population studied: Hospital (H)/Community (C). ^b^ Percentage of positivity for AmpC among all isolates evaluated in the study. ^c^ Percentage global positivity that includes species other than *E. coli* and *K. pneumoniae* and/or does not differentiate between cAmpC-BL and pAmpC-BL. PCR: polymerase chain reaction; WGS: whole genome sequencing; ECI: *E. coli* isolates; 3GCR: third-generation cephalosporin-resistant; EI: *Enterobacteriaceae* isolates; MDR: multidrug resistant.

PRESENCE IN AFRICA (Table 4). Reports on the prevalence of pAmpC-BL have varied widely among African countries. *E. coli* percentages have ranged from 0.50 in Tanzania (2016) [56] and 0.59% in Morocco (2013) [57] to 14.68% in Egypt (2014) [58], with reports of 4.23% in Nigeria (2014) [59] and 10.86% in Mozambique (2021) [60]. Likewise, the percentages of *K. pneumoniae* isolates have ranged between 0.88 in Morocco (2013) [57] and 3.97% in Libya (2017) [61]. Overall, the highest prevalence rates of pAmpC-BL have been described in Uganda (2014) [60], with a rate of 39.6% among FOX-resistant isolates; in Egypt (2014) with 18.8% [58]; and in Nigeria (2014) with 11.23% [59].

**Table 4:** Epidemiology of pAmpC-BLs in Africa (2010–2020).

Author (Reference)	Year of Population Study	Year of Publication	Country of Target Population	Population (H/C) ^a^	Specific Conditions	n	AmpC (%) ^b^		Global ^c^	Genetic Identification	Most Frequent AmpC Enzymes
							*E. coli*	*K. pneumoniae*			
Ogbolu et al. [62]	2005–2007	2011	Nigeria	H	EI	134	-	-	4.50	PCR/WGS	DHA-1
Barguigua et al. [63]	2010	2013	Morocco	C	ECI	1,174	0.59	-	-	PCR/WGS	CIT group
Barguigua et al. [57]	2010–2011	2013	Morocco	C	KPI	453	-	0.88	-	PCR/WGS	EBC group
Yusuf et al. [59]	*Data not available*	2014	Nigeria	H/C	EI	543	4.23	3.50	11.23	-	-
Helmy et al. [58]	2011–2012	2014	Egypt	H	EI	143	14.68	2.09	18.18		CIT group
Nakaye et al. [64]	2013	2014	Uganda	H	3GCR	293	-	-	39.60	PCR	EBC group
Gharout-Said et al. [65]	2005–2010	2015	Algeria	H	EI	922	-	-	1.60	PCR/WGS	CMY-4
Chérif et al. [66]	2006–2009	2015	Tunisia	H	EI	11,393	-	-	0.59	PCR/WGS	CMY-2
Tellevik et al. [56]	2010–2011	2016	Tanzania	H/C	EI	603	0.50	-	-	PCR/WGS	CMY-2
Zorgani et al. [61]	2013–2014	2017	Libya	H	EI	151	1.98	3.97	5.96	PCR	CIT group
Tanfous et al. [67]	2002–2011	2018	Tunisia	H	KPI	128	-	2.30	-	PCR/WGS	CMY-4
Tanfous et al. [68]	2002–2013	2018	Tunisia	H	ESBL	128	-	2.34	-	PCR/WGS	CMY-4
Rensing et al. [69]	2013	2019	Egypt	H/C	EI	225	1.45	0.97	2.91	PCR	CIT group
Mohamed et al. [70]	2018	2020	Egypt	C	EI	440	2.04	2.04	4.09	PCR/WGS	DHA-1
Estaleva et al. [60]	2015	2021	Mozambique	H/C	ECI	230	10.86	-	-	PCR/WGS	FOX/MOX

^a^ Type of population studied: Hospital (H)/Community (C). ^b^ Percentage positivity for AmpC among all isolates evaluated in the study. ^c^ Percentage global positivity that includes species other than *E. coli* and *K. pneumoniae* and/or does not differentiate between cAmpC-BL and pAmpC-BL. PCR: polymerase chain reaction; WGS: whole genome sequencing; ECI: *E. coli* isolates; 3GCR: third-generation cephalosporin-resistant; EI: *Enterobacteriaceae* isolates; KPI: *K. pneumoniae* isolates; ESBL: extended spectrum beta-lactamase.

PRESENCE IN ASIA, OCEANIA, AND THE MIDDLE EAST (Table 5). These regions have reported the highest prevalence rates of pAmpC-BL isolation. The rate of *K. pneumoniae* ranges from 0.01% in Japan (2010) [21] to a very high rate of 44.95% in a Hong Kong study (2016) [71]. Elevated rates have also been described in India (2010 and 2012) [72,73] with 13.14% and 13.27% positivity, respectively; Pakistan (2013) [74], with 12.37% positivity among isolates with ESBL phenotype; and China (2015) [75], with 31.50% positivity among multi-drug resistant *K. pneumoniae* isolates. Positivity rates in *E. coli* have ranged from 0.07% in Japan (2010) [21] to India (2010 and 2012) [72,73] with 24.57% and 24.89% positivity, respectively.

**Table 5:** Epidemiology of pAmpC-BLs in Asia, Oceania, and the Middle East (2010–2020).

Author (Reference)	Year of Population Study	Year of Publication	Country of Target Population	Population (H/C) ^a^	Specific Conditions	n	AmpC (%) ^b^		Global ^c^	Genetic Identification PCR/WGS	Most Frequent AmpC Enzymes
							*E. coli*	*K. pneumoniae*			
Yoo et al. [76]	2008–2009	2010	South Korea	H	EI	276	1,81	16.66	-	PCR	DHA-1
Yamasaki et al. [21]	2002–2008	2010	Japan	H/C	EI	22,869	0.07	0.01	0.13	PCR/WGS	CMY-2
Singtohin et al. [77]	2005–2006	2010	Thailand	H	EI	2,712	1.62	0.29	1.91	PCR	CMY-2
Mohamudha et al. [72]	2008	2010	India	H	EI	175	24.57	13.14	44.57	-	-
Mohamudha et al. [73]	2009–2010	2012	India	H	EI	241	24.89	13.27	38.17	PCR	DHA-1
Manoharan et al. [78]	2007–2008	2012	India	H	3GCR	312	-	-	15.38	PCR	CIT group
Matsumura et al. [79]	2010	2012	Japan	H	ECI	1,327	1.73	-	-	PCR/WGS	CMY-2
Gupta et al. [80]	2008–2009	2012	India	H	KPI	100	-	32	-	PCR	CMY-2
Sasirekha et al. [81]	2008	2012	India	H	EI	90	4.44	3.33	7.77	-	-
Shafiq et al. [82]	2008	2013	Pakistan	H/C	ESBL	511	7.97	12.37	-	-	-
Azimi et al. [83]	2013	2015	Iran	H	KPI	303	-	1.60	-	PCR/WGS	CMY-
Hou et al. [75]	2011	2015	China	H	KPI- MDR		-	31.50	-	PCR	DHA-
Liu et al. [84]	2012	2016	China	H	ECI	96	12.50	-	-	PCR	DHA-1
Liu et al. [85]	2012	2016	China	H	KPI	130	-	10.80	-	PCR/WGS	DHA-1
Ghosh et al. [86]	*Data not available*	2016	India	H	EI	148	16.89	-	-	PCR/WGS	CMY-2
Luk et al. [71]	2004–2008	2016	Hong Kong	H	KPI	109	-	44.95	-	PCR	DHA-1
Sadeghi et al. [87]	2014	2016	Iran	H	EI	307	-	-	21.50	PCR/WGS	CMY-2
Baljin et al. [88]	2014	2016	Mongolia	H	EI	478	0.41	-	-	PCR/WGS	CMY-2
Noguchi et al. [89]	2011–2012	2017	Japan	H	EI	316	0.63	0.95	-	PCR/WGS	DHA-1
Khurana et al. [29]	2013–2015	2017	India	H	GNB	761	0.52	-	-	PCR	FOX-1/FOX-5b
Abdalhamid et al. [90]	2015	2017	Saudi Arabia	H	EI	3,625	-	-	1	PCR/WGS	CMY-2
Harris et al. [22]	2014–2015	2018	Australia, New Zealand, Singapore	H	3GCR	30	17.10	-	-	PCR/WGS	CMY-2
Nishimura et al. [91]	2005–2011	2018	Japan	H	EI	8,299	0.54	-	1.75	PCR/WGS	CIT group
Kim et al. [92]	2007–2016	2019	South Korea	H	ECI	1,047	1.52	-	-	PCR/WGS	DHA-1
Rizi et al. [93]	2018	2020	Iran	H	EI	602	-	-	9.30	PCR	CMY-2
Shrestha et al. [94]	2013–2014	2020	Nepal	H/C	ECI	2,661	9.86	-	-	-	-
Aryal et al. [95]	2017–2018	2020	Nepal	H	GNB	226	-	-	40.26	PCR	CIT group
Bala et al. [96]	2018	2020	India	H	ECI	470	11.10	-	-	PCR	CIT group

^a^ Type of population studied: Hospital (H)/Community (C). ^b^ Percentage positivity for AmpC among all isolates evaluated in the study. ^c^ Percentage global positivity that includes species other than *E. coli* and *K. pneumoniae* and/or does not differentiate between cAmpC-BL and pAmpC-BL. PCR: polymerase chain reaction; WGS: whole genome sequencing; ECI: *E. coli* isolates; 3GCR: third-generation cephalosporin-resistant; EI: *Enterobacteriaceae* isolates; KPI: *K. pneumoniae* isolates; ESBL: extended spectrum beta-lactamase; GNB: Gram-negative bacilli; MDR: multidrug resistant.

#### 3.1.2. Phenotypic Detection Methods

Since the discovery of AmpC type resistances several decades ago, the approach to their detection has been controversial, attributable to the lack of clear guidelines from CLSI or EUCAST. This has led to an underdiagnosis of AmpC-BLs, contributing to underestimation of the prevalence and global spread of this type of resistance. In 2018, Conejo et al. (2018) called for an improvement in the phenotypic detection of AmpC-BLs in Spanish clinical laboratories [97]. The ability of a laboratory to detect both types of AmpC resistance is essential, and the detection of pAmpC-BLs is of vital epidemiological importance, given their transmission and dissemination capacity and their association with outbreaks of community and nosocomial infections [17,98]. AmpC-BL detection is especially difficult in microorganisms that can produce chromosomal and plasmid AmpC-BL (e.g., *E. coli*). In these cases, the presence of a plasmid needs to be investigated to monitor their spread more closely [99] and address the clinical, therapeutic, epidemiological, and organizational repercussions, including the isolation of infected patients. For the laboratory detection of these resistances, the presence of pAmpC-BLs should be surveilled in species without chromosomal AmpCs that have proven able to disseminate these enzymes, mainly *K. pneumonia* [100]. The behavior of antibiotics against pAmpC-BL-producing microorganisms is characterized by a decreased susceptibility to oxyimino-cephalosporins (e.g., CTX or CAZ) and methoxy-cephalosporins (e.g., FOX), and a susceptibility to fourth-generation cephalosporins (e.g., FEP) [17,101]. Therefore, isolates showing some of these characteristics in the antibiogram should be suspected of pAmpC-BL production when there is no other apparent cause. However, there have been multiple reports of AmpC-producing bacteria that appeared susceptible to both oxyimino-cephalosporins and FOX in the antibiogram [18,19,24,97,99,102,103,104,105,106]. This may be attributable to a so-called “inoculum effect” (susceptibility in vitro at low microorganism concentrations but inefficacy in vivo) [24,106] or to the conventional consideration of these isolates as susceptible to cephalosporins in vitro according to now-outdated CLSI or EUCAST cutoff points [103,106]. It should be borne in mind that pAmpC-BL-producing isolates occasionally present with more than one beta-lactamase and are multidrug resistant [4,75,94,101]. The presence of AmpC can mask the coexistence of ESBL, hampering differentiation of the two resistances [22,79,99,105,107,108], because the two enzyme groups are hydrolytically very similar, except that AmpC-BL is not inhibited by CLAV, with reports of an increased cephalosporin MIC in its presence [107].

**Figure 1:**
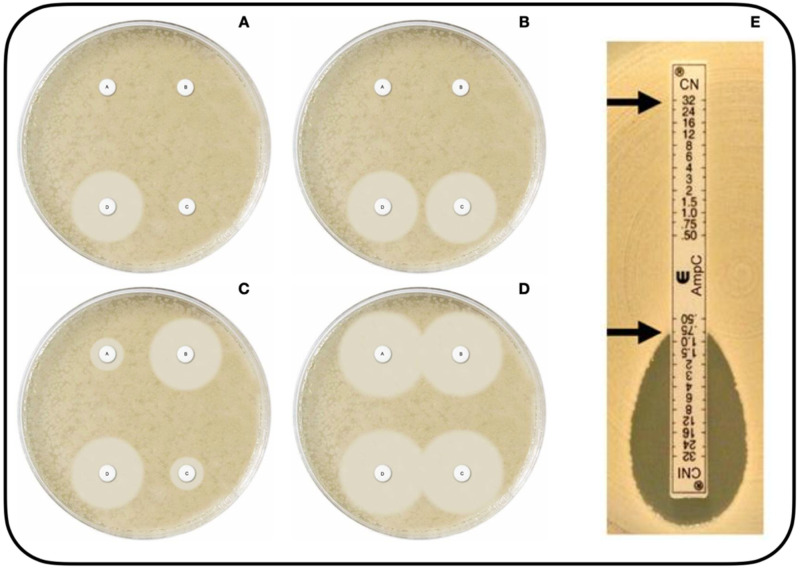
Phenotypic methods of AMPc beta-lactamase detection (Available online: https://mast-group.com/uk/products/amr/antibiotic-resistance-detection-sets/d68c/ (accessed on 25 October 2021)). Mast Disk test, with four disks [123]: Disk A(Cefpodoxime), disk B (Cefpodoxime + ESBL inhibitor), disk C (Cefpodoxime + AmpC inhibitor), disk D (Cefpodoxime + AmpC inhibitor + ESBL inhibitor). (**A**): positive result for AmpC production alongside ESBL production; (**B**): positive result for AmpC production; (**C**): positive result for ESBL production; (**D**): no AmpC or ESBL production. Image (**E**) depicts the AmpC E-test with cefotetan (CN) and cefotetan with cloxacillin (CNI), showing a positive result for the presence of AmpC beta-lactamase with a major reduction in MIC at the CNI end [124].

There appears to be a consensus that AmpC-BL screening should consider reduced susceptibility to cephamycins and/or oxyimino-cephalosporins, specifically FOX resistance, alongside the occasional resistance to conventional inhibitors such as CLAV. However, CLAV resistance should be evaluated with caution, given that some isolates may appear susceptible [4]. Accordingly, Meini et al. (2019) proposed routine laboratory FOX screening in laboratories for the detection of AmpC-BLs, proposing FOX MIC > 8 mg/L combined with resistance to CTX and/or CAZ as phenotypic indicator of the presence of pAmpC-BL [17,30]. EUCAST recommends investigating the presence of AmpC when the aforementioned antibiogram results are obtained [98]. Other authors have also recommended screening for susceptibility to FEP, to which AmpC-BLs are susceptible [109]. However, it should be borne in mind that some *E. coli* isolates can have reduced susceptibility to FEP (MICs ranging from 0.5 to 12 μg/mL), being known as *E. coli* producing extended-spectrum AmpC (ESAC) beta-lactamases [110]. Other drawbacks of phenotypic tests are their incapacity to differentiate pAmpC-BL producers if they present inducible cAmpC-BL. Known limitations of phenotypic tests also include confluence with other resistance mechanisms such as ESBLs and porin loss [111]. Specific phenotypic tests are based on the detection of AmpC enzyme hydrolytic activity or utilization of AmpC inhibitors and their capacity to suppress beta-lactamase expression [112]. Various phenotypic methods have been proposed for detecting AmpC-producing microorganisms:

- Disk approximation method. This technique is employed to detect inducible AmpC-BLs. In the case of pAmpC-BLs, it would be valid for the AmpCs of DHA-1, DHA-2, ACT-1, and CMY-13 families. Two disks are used, one with a substrate antibiotic such as an oxyimino-cephalosporin (e.g., CAZ) or piperacillin/tazobactam, and the other with an inducer antibiotic (e.g., FOX, CLAV, or imipenem, etc.). The microorganism produces an inducible BL if the substrate antibiotic inhibition halo is reduced in the area close to FOX [113].
- Methods with AmpC-specific inhibitors. CLX and boronic acid (BA), and their derivatives, have proven to be the most active and effective commercially available inhibitors to detect AmpC-BLs [105,113,114,115], with CLX being more specific [114,116]. The combination of CTT with other inhibitors, such as Ro48-1220 and LN-2-128 [117,118] or Syn2190 [101,118], especially Syn2190, have demonstrated high sensitivity and specificity to detect AmpC-producing microorganisms; however, they are not commercially available. The main methods include:
  (a) *Double-disk potentiation methodwith BA or CLX* [98,113,119,120,121]. Cephalosporin disks, especially CTT, FOX, CAZ, or CTX, are used alone and supplemented with BA or CLX, obtaining a positive result when the difference in inhibition halo in the disk with inhibitor is >5 mm.
  (b) *Double-disk synergy method with double BA* [113,115,120] *or CLX* [3,28,120]. A BA or CLX disk is placed with a CAZ disk and CTX disk on both sides. The test is positive when the inhibition halo is distorted (augmented).
  (c) *AmpC detection disks*. This technique, described by Black et al. (2005) [122], uses Tris-EDTA to permeabilize the bacterial membrane and release beta-lactamases. The bacterium suspected of producing pAmpC-BL (study bacterium) is added to the AmpC disks, which contain Tris-EDTA. The medium is inoculated with an isolate known to be susceptible to FOX (control bacterium), and a FOX disk with AmpC disks (containing the studied bacterium) is placed on both sides. A flattening of the FOX inhibition halo indicates antibiotic inactivation (i.e., the presence of AmpC enzyme released into the medium from the studied bacterium) and therefore a positive result for the presence of pAmpC-BL [28,113,122].
  (d) *Three-dimensional method*. A FOX disk is placed in an agar plate inoculated with a strain susceptible to this antibiotic. An incision is made in the agar near the disk for inoculation with the microorganism under study. The result is positive when the inhibition halo is flattened, which is caused by the growth of the AmpC-producing microorganism [73,113,121].
  (e) *Mast disks* (MastDics^®^ Combi AmpC and ESBL Detection Set, Merseyside, UK) (Figure 1A–D) [123]. This technique, which can be used to detect both AmpC and ESBL, utilizes cefpodoxime disks, alone and combined with AmpC inhibitor and/or ESBL inhibitor. The result is positive when the difference in halo diameter between disks with *versus* without inhibitor is >5 mm [112].
  (f) *E-test^®^ AmpC* (Biomérieux SA, 69280, Marcy-l´Etoile, France) (Figure 1E) [124]. This test utilizes strips impregnated with CTT at increasing concentrations on both sides, with the presence of CLX on only one side. The result is positive if there is a CTT MIC reduction of at least three dilutions or deformation of the ellipse in the presence of CLX [52,112,121].
  (g) *ESBL + AmpC Screen ID Kit* (ROSCO, Albertslund, Denmark). Similar concept to the Mast disks referred to in point e). In this case, the cephalosporin is cefotaxime [125,126].

#### 3.1.3. Genotypic Detection Methods

Multiplex PCR has been considered the gold standard technique for the detection of pAmpC-BL since its development by Pérez-Pérez et al. in 2002. It permits the detection and differentiation of plasmid AmpC families and of their chromosomal or plasmid origin (valuable for species with both types of AmpC, such as *E. coli*) [16]. Modifications have been introduced since its invention, including simple PCRs that offer the same diagnostic value and may even be used for screening (Dallenne et al., 2009] [127]; real-time PCR (Brolund et al., 2010) [128] for rapid detection, using affordable reagents; and, more recently, multiplex real-time PCR (Chavda et al., 2016) [129], which offers the identification of AmpC along with other types of resistance, again in a relatively short time. Voets et al. (2011) [130] developed a novel multiplex PCR capable of identifying up to 25 types of beta-lactamases in one amplification reaction (including ESBL, pAmpC, carbapenemases, etc.), and Geyer et al. (2012) [131] obtained 100% sensitivity and specificity using multiplex real-time PCR. Liu et al. (2015) [132] created a rapid and more cost-effective technique that allowed 96 samples to be tested within 2 h using standard real-time PCR equipment.

It was initially considered impossible for a single isolate to express more than one pAmpC-BL for two reasons: AmpC detection is not sufficiently precise, and the amount of AmpC in the bacterium is too limited for it to be a viable pathogen [16,132]. However, it is now considered possible, given that various authors have reported the finding of more than one *ampC* gene in the same isolate [45,52,61,66,93].

#### 3.1.4. Behavior of *Enterobacteriaceae* with AmpC-BL in ChromID^®^ ESBL Medium, Using Disk Diffusion Test with CLX Disks

**Figure 2:**
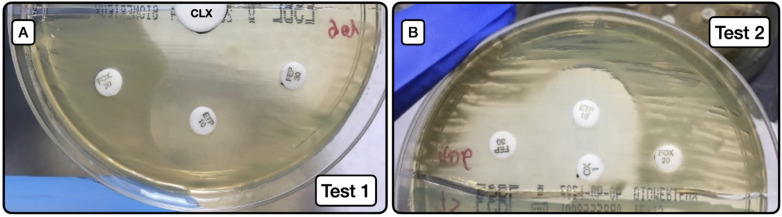
Screening method for the phenotypic detection of AmpC. (**A**): Image of test 1, depicting the synergy between the large white disk with CLX (20 µL at concentration of 50 mg/mL) and ETP and FEP susceptibility halos. (**B**): Image of test 2, depicting the same synergy phenomenon as in A, but in this case between the oxacillin disk with CLX (10 µL at concentration of 50 mg/mL) and ETP and FEP disks. CLX (cloxacillin); ETP (ertapenem); FEP (cefepime); FOX (cefoxitin).

The behaviors of test 1 **(**Figure 2A) and test 2 (Figure 2B) are exhibited in Table 6, Table 7 and Table 8.

Screening test 1 (Figure 2A) to detect AmpC producers, either as sole resistance mechanism or in combination with ESLB production, had a sensitivity of 31.3%, specificity of 100%, positive predictive value of 100%, and negative predictive value of 88.7%. When the test was applied to detect AmpC producers alone (not combined with ESBL), the sensitivity was 55.6%, the specificity 100%, positive predictive value 100%, and negative predictive value 95.6%.

Screening test 2 (Figure 2B) to detect AmpC producers, either as a sole resistance mechanism or in combination with ESLB production, had a sensitivity of 62.5%, specificity of 100%, positive predictive value of 100%, and negative predictive value of 93.5%. When the test was applied to detect AmpC producers alone (not combined with ESBL), the sensitivity was 88.9%, specificity 100%, positive predictive value 100%, and negative predictive value 98.9%.

Comparison of test results using the Youden index showed that test 2 had greater predictive capacity. Notably, a Youden index of 0.889 was obtained for the detection of AmpC-producing isolates without the presence of ESLB producers. 

## 4. Discussion

This review reveals increasing research interest in pAmpC-BLs over the past few years. Numerous scientific publications have described their detection in the environment, including aquatic media, in food production, and in samples from animals and humans [133].

The reviewed data show that the overall prevalence of pAmpC-BL is higher in the region comprising Asia, Oceania, and the Middle East than in the rest of the world, especially India (44.47% in 2012) [72], Nepal (40.26% in 2020) [95], and Iran (20.50% in 2020) [87]. The lowest prevalence has been reported in Europe, followed by America. Nevertheless, it should be taken into account that some studies did not differentiate between cAmpC-BL and pAmpC-BL, and may therefore have overestimated the prevalence of pAmpC-BL. Prevalence data also vary markedly according to the methodology applied, which may at least in part explain the wide differences reported among countries [51,61,72,86,91,93] and among regions, bacterial species, and study dates [121]. For example, the percentage of pAmpC-BL positivity described by Shrestha et al. (2020) in Nepal was very high (40.26%) because they studied a sample of gram-negative bacilli, in general, including non-fermenting gram-negative bacilli [95].

However, there can be no doubt that the worldwide prevalence of pAmpC-BL has increased, rising globally in the USA from 1.3% (2016) [32] to 3.42% (2019) [52] and in Egypt from 2.91% in *E. coli* isolates in 2019 [69] to 4.09% in 2020 [70]. In India, the prevalence in *E. coli* isolates increased from 4.44% in 2012 [81] to 11.10% in 2020 [96]. Major efforts are needed to control the spread of this type of beta-lactamase and avoid hospital/community outbreaks and endemic dissemination, such as that observed between 2010 and 2012 in Hungary, the first European country with endemic dissemination of a pAmpC-BL, specifically DHA-1 plasmid [134].

In Spain, the prevalence of pAmpC-BL in *E. coli* has been reported in various studies as 0.69% (2013) [43], 0.78% (2014) [45], 1.1% (2016) [49], and 2.02% (2016) [50], and the prevalence in *K. pneumoniae* was 1.02% in 2013 [43]. One of the studies in 2016 (50) suggested that pAmpC-BLs are the main mechanism of AmpC production in Spain.

Although the most frequent type of plasmid worldwide is CMY-2 of the CIT family, a high percentage of DHA-1 isolates has been reported in Asia, especially in China, where DHA-1 is the most commonly isolated plasmid [28,75,84,132]. DHA-1 is also the most frequent plasmid in *K. pneumoniae* isolates, whereas CMY-2 is the most common in *E. coli* isolates, as noted above. A striking finding is the predominance of FOX/MOX plasmids recently observed in Mozambique (2021), surpassing both CMY-2 and DHA-1 plasmids [60].

The presence of pAmpC-BL should be suspected when isolates have a BL resistance pattern that differs from their wild phenotype [121], given the well-documented validation of AmpC-BL screening detecting a reduced susceptibility to FOX [17]. Importantly, however, this screening procedure cannot detect AmpC-BLs of the ACC family, which are incapable of hydrolyzing FOX, and microorganisms that express these appear as susceptible to it in the antibiogram [5,17,103,114,116]. Reduced susceptibility to FOX may be due to not only AmpC-BL production but also to a reduced permeability of the external bacterial membrane [20,106]. Among the phenotypic methods reviewed, the “AmpC disk method” is a highly sensitive and specific method that can differentiate resistance to FOX due to the presence of AmpC-BL from that caused by a reduction in external membrane permeability [122], which cannot be achieved by the BA disk synergy method [20].

Phenotypic techniques are simple, generally inexpensive, rapid, and readily interpreted [112,114], favoring their incorporation in routine laboratory analyses to detect possible resistance-producing microorganisms. However, they are not capable of differentiating between pAmpC-BLs and cAmpC-BLs [3,17,19,98,115,116,119] or between different pAmpC-BL families [3]. Hence, a study of *E. coli* based on phenotypic methods alone would have high sensitivity but low specificity, because it may detect many *E. coli* isolates that are hyperproducers of chromosomal AmpC [98]. In *Klebsiella* spp., which lacks cAmpC-BL, a positive phenotypic result for AmpC would be confirmatory because it would imply the presence of pAmpC-BL [120,121]. One limitation of phenotypic methods using BA and its derivatives is that dimethyl sulfoxide is usually employed for their dilution, and this toxic substance needs to be handled with caution. However, their dilution in distilled water has also been described, resolving this problem [113,135]. A further limitation is that BA is not specific for AmpC, and positive results can result from the presence of ESBL and carbapenemases [121].

Few recommendations have been published on the application of these techniques, which require clear guidelines for their interpretation [104]. A good approach to the detection of AmpC may be to apply lower cutoff points than those habitually used for CAZ, ceftriaxone, and CTX. For instance, Agyekum et al. (2016) [136] described all isolates with CTX MIC > 1 mg/L as non-susceptible. Another shortcoming of phenotypic methods is that they are not all equally effective at detecting all AmpC families [20,137], and Ingram et al. (2011) found that inhibitors such as CLX or BA are more sensitive to DHA than CMY [114].

Proposed phenotypic methods that have demonstrated greatest diagnostic usefulness are those based on inhibitors. Thus, acceptable sensitivity and specificity values (>90%) are obtained using the double-disk synergy method with CLX + FOX [20,30,114] and the AmpC disk method [114,122]. The AmpC E-test has obtained the worst results, possibly because it contains CTT, showing lower sensitivity but higher specificity to detect AmpC [114].

The rapid fluorogenic method (1.5 h) recently developed by Park et al. (2020) has demonstrated high sensitivity and specificity to detect pAmpC-BL. It utilizes an antimicrobial bound to a fluorogenic substance that emits fluorescence in the presence of a beta-lactamase capable of its hydrolyzation. This method can also be combined with direct diagnostic techniques (Vitek2^®^ or MALDI-TOF^®^) for the rapid detection of the bacterial species [138]. This represents an advancement in the development of novel methods for pAmpC-BL detection.

The coexistence of ESBL and AmpC poses a diagnostic challenge, given that ESBL resistance may go unnoticed in AmpC-producing organisms by presenting this resistance to CLAV. It is also possible that FOX resistance may be produced by a combination of ESBL production and reduced external membrane permeability [120]. A possible solution to the diagnostic challenge posed by the coexistence of ESBL and AmpC is to include FEP, which is not affected by the presence of AmpC, in ESBL screening along with CLAV [17,99]. In cases of ESBL and AmpC coexistence, BA is a more diagnostically valuable inhibitor [128]. Song et al. (2007) [139] modified the CLSI ESBL detection technique, which uses CLAV, by adding BA to CTX or CAZ disks and to CTX/CAZ disks with CLAV, producing CTX/CAZ+BA disks and CTX/CAZ+CLAV+BA disks. An increase of ≥3 mm in the halo of the CTX/CAZ+CLAV+BA *versus* CTX/CAZ+BA disk is considered positive for ESBL [113,135,140].

Genotypic methods are considered the gold standard techniques for the detection of AmpC resistances [3,30,114] and are able to differentiate between chromosomal and plasmid AmpC-BLs [98]. However, most of them are expensive, technically complex, and time-consuming methods that are mainly used in research and are reserved for doubtful cases in a clinical setting (e.g., *E. coli* isolates) [140]. Another major drawback is that they can detect *ampC* genes that are already known but not new mutations or AmpC families [16,98,112,115,132].

**Figure 3:**
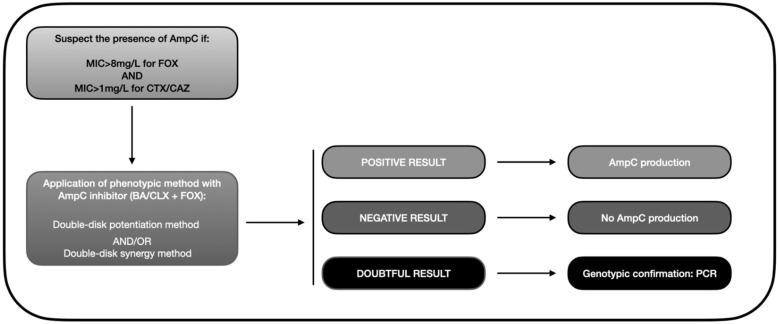
Diagnostic algorithm for the phenotypic detection of AmpC-BL (based on data from the review). MIC: minimum inhibitory concentration; FOX: cefoxitin; CAZ: ceftazidime; BA: boronic acid; CLX: cloxacillin; PCR: polymerase chain reaction.

Based on the data gathered in this review, we developed an algorithm similar to that depicted in Figure 3. Accordingly, the presence of AmpC-BL should be suspected when there is resistance to cephamycins (MIC > 8 mg/L for FOX) and oxyimino-cephalosporins (pattern of resistance to CTX or CAZ). This helps avoid a search for AmpC-BL in ESBL-producing bacteria, which would meet the second but not the first criterion because they are susceptible to FOX. A phenotypic method should then be applied to confirm the presence of AmpC, with the double-disk synergy test being the most highly recommended approach. Finally, a PCR or a genotypic analysis should be carried out in doubtful cases to verify the presence/absence of AmpC-BL encoding genes, bearing in mind that some isolates present positivity in the double-disk method that even PCR cannot detect [102,135].

Regarding our screening proposal (see Section 2.2) for the phenotypic detection of AmpC-producing *Enterobacteriaceae*, test 2 obtained the best results. In comparison, Reuland et al. [30] obtained 91% sensitivity and 96% specificity using the double-disk synergy method with CLX in 66 isolates with reduced susceptibility to FOX and third-generation cephalosporins, and 85% sensitivity and 95% specificity when they applied the same method but with BA [30]. The use of CTT disks alone, and with the addition of BA as phenotypic confirmation, was recommended in a study with 635 isolates of *Enterobacteriaceae* not susceptible to FOX (MIC ≥ 32 mg/dL) [141]. In another study using the double-disk synergy method to test 255 isolates, the addition of CLX to a FOX disk, obtaining the best predictive values when the halo increase ≥4 mm, was considered positive, achieving 95% sensitivity and 95% specificity [20]. Finally, Polsfuss et al. used the double-disk synergy method in 305 isolates and described 97.2% sensitivity and 100% specificity [114]. With regard to other techniques, Black et al. used the AmpC disk to screen 140 isolates not susceptible to FOX and obtained 100% sensitivity and 98% specificity [122]. Ingram et al. compared different screening and confirmatory methods in a study of 246 isolates, concluding that the screening method with the AmpC disk obtained the best result, offering 95% sensitivity and 98% specificity [109]. In a sample of 125 pAmpC-BL-positive isolates, the CTT/CXT E-Test showed 98.6% sensitivity and 35.4% specificity, while the AmpC disk method obtained slightly lower sensitivity (96%) but higher specificity (58%) values [44]. Hence, the results achieved with test 2 are comparable to the best results described for screening phenotypic tests, with the added advantage of integrating this fourth disk in the screening test with FEP, FOX, and ETP disks, previously proposed by our group for the detection of microorganisms with ESBL and/or carbapenemase [9].

Test 2 yields lower values in the presence of ESBL. As noted above, the coexistence of AmpC and ESBL production hampers the phenotypic detection of both resistance mechanisms, and phenotypic methods are recommended with the addition of CLAV (AmpC inducer and ESBL inhibitor) and even genotypic methods for a definitive diagnosis [17]. Song et al. used BA as AmpC inhibitor in their study of 182 isolates, comparing CTX/CA/BA disks with CTX and/or CAZ/CA/BA with CAZ disks as well as CTX/CA/BA disks with CTX/BA disks and/or CAZ/CA/BA disks with CAZ/BA disks, reporting that both approaches markedly improved sensitivity and specificity values in comparison to the utilization of CA alone [139]. However, a review proposed the double-disk synergy method using CTX and CAZ with and without the addition of CLX as the optimal phenotypic confirmation test for AmpC in the presence of ESBL [119]. The diagnostic usefulness of test 2 in these isolates is enhanced by increasing the amount of CLX on the oxacillin disk.

### Limitations

Studies with larger samples of AmpC-producing isolates are required to obtain more accurate predictive values. In common with other phenotypic techniques, our method cannot differentiate between the presence of plasmid or chromosomal AmpC except when the isolate is known to produce plasmid AmpC producer alone, as in the case of *K. pneumoniae*. In addition, the detection of pAmpC-BL is hampered by confluence with other resistance mechanisms such as ESBL production, porin loss, or *E. coli* producing ESAC beta-lactamases. Reference methods used for the detection of AmpC producers were the cefotetan/cefotetan-CLX E-Test and an increased CTX and CAZ MIC in the presence of CLAV, because the means required for genotypic identification of the AmpC resistance mechanism were not available. 

## 5. Conclusions

There has been an increase in pAmpC-BL-producing isolates over the past 10 years, especially in the Asian continent, and CMY-2 producers are the most frequently responsible. Prevention of their spread requires the implementation of routine surveillance procedures that combine phenotypic and genotypic approaches (multiplex PCR). Among phenotypic screening methods, double-disk synergy and AmpC disk methods can be especially recommended for their predictive capacity. Our proposed screening method, which involves the addition of CLX on an oxacillin disk, is an easy-to-use and inexpensive test for the detection of AmpC-producing isolates, especially when there is no other resistance mechanism. Moreover, it can be combined on a single plate with a screening method for the detection of *Enterobacteriaceae* with ESBL and/or carbapenemases through the addition of FOX, FEP, and ETP disks. 

## Figures and Tables

**Table 1:** Isolation of the first pAmpC-BLs (modified by Jacoby, 2009) [3].

pAMPC-BL Enzyme	Country of Discovery	Year of Isolation	First Species in Which It Was Isolated	Chromosomal Origin Species	% Similarity (with Respect to the Chromosomal Gene)
CMY-1	South Korea	1989	*K. pneumoniae*	*A. hydrophila*	82
CMY-2	Greece	1996	*K. pneumoniae*	*C. freundii*	96
MIR-1	USA	1990	*K. pneumoniae*	*E. cloacae*	99
MOX-1	Japan	1993	*K. pneumoniae*	*A. hydrophila*	80
LAT-1	Greece	1993	*K. pneumoniae*	*C. freundii*	95
FOX-1	Argentina	1994	*K. pneumoniae*	*A. caviae*	99
DHA-1	Saudi Arabia	1997	*S. enteriditis*	*M. morganii*	99
ACT-1	USA	1997	*K. pneumoniae*	*E. asburiae*	98
ACC-1	Germany	1999	*K. pneumoniae*	*H. alvei*	99
CFE-1	Japan	2004	*E. coli*	*C. freundii*	99

pAmpC-BL: plasmid AmpC beta-lactamases.

**Table 6:** Detection of isolates with AmpC using test 1.

	**Test 1 Negative**	**Test 1 Positive**	**Total**
No AmpC	86	0	86
AmpC ± ESBL	11	5	16
	97	5	102
	**Test 1 Negative**	**Test 1 Positive**	**Total**
No AmpC	86	0	86
AmpC alone	4	5	9
	90	5	95

**Table 7:** Detection of isolates with AmpC using Test 2.

	**Test 2 Negative**	**Test 2 Positive**	**Total**
No AmpC	86	0	86
AmpC ± ESBL	6	10	16
	92	10	102
	**Test 2 Negative**	**Test 2 Positive**	**Total**
No AmpC	86	0	86
AmpC alone	1	8	9
	87	8	95

ESBL: extended-spectrum beta-lactamase.

**Table 8:** Usefulness of screening methods with tests 1 and 2.

(CI 95%)	Test 1		Test 2	
**Indicators**	**AmpC ± ESBL**	**AmpC Alone**	**AmpC ± ESBL**	**AmpC Alone**
Prevalence	15.7%	9.5%	15.7%	9.5%
Sensitivity	31.3% (14.2–55.6)	55.6% (26.7–81.1)	62.5% (38.6–81.5)	88.9% (56.5–98)
Specificity	100% (95.7–100)	100% (95.7–100)	100% (95.7–100)	100% (95.7–100)
PPV	100% (56.6–100)	100 (55.6–100)	100% (72.2–100)	100% (67.6–100)
NPV	88.7% (80.8–93.5)	95.6% (89.1–98.3)	93.5% (86.5–97)	98.9% (93.8–99.8)
Validity Index	89.2% (81.7–93.9)	95.8% (89.7–98.4)	94.1% (87.8–97.3)	98.9% (94.3–99.8)
Youden’s Index	0.313	0.556	0.625	0.889

PPV: positive predictive value; NPV: negative predictive value; ESBL: extended-spectrum beta-lactamase; CI: confidence interval.

## Data Availability

The data presented in this study are available in the main text.
